# The diguanylate cyclase CrdA contributes to cell adhesion and gut colonization by switching motility and sessile state of *Cronobacter malonaticus*

**DOI:** 10.3389/fmicb.2026.1922582

**Published:** 2026-09-25

**Authors:** Jia Jia, Siqi Li, Xinlu Yu, Feiyan Jiao, Yufei Han, Jing Zhang, Bingxin Liu, Meiling Jiang, Ge Yun, Dan Liu, Yue Ma, Jiping Yu, Mingming Song, Shuwei Yan, Haifeng Wang, Junlong Zhao, Yuanyuan Wang, Gukui Chen

**Affiliations:** 1 Department of Laboratory, Northwest University First Hospital, Xi’an, China; 2 Key Laboratory of Resource Biology and Biotechnology in Western China, Ministry of Education, Faculty of Life Science and Medicine, Northwest University, Xi’an, China; 3 School of Medicine, Northwest University, Xi’an, China; 4 Department of Laboratory, Xi’an No.3 Hospital, The Affiliated Hospital of Northwest University, Xi’an, China; 5 College of Medical Technology, Shaanxi University of Chinese Medicine, Xianyang, China

**Keywords:** bioflim, c-di-GMP, cell adhesion, *Cronobacter malonaticus*, diguanylate cyclases, gut colonization, motility

## Abstract

*Cronobacter malonaticus* is an emerging foodborne pathogen posing severe threats to vulnerable populations. Despite the established role of the ubiquitous second messenger cyclic diguanylate (c-di-GMP) in bacterial virulence, the specific diguanylate cyclases (DGCs) that orchestrate pathogenicity in *C. malonaticus* are unknown. Here, we systematically characterized 13 putative DGCs in *C. malonaticus* and identified CrdA as a pivotal regulator. We demonstrated that CrdA reciprocally inhibits swimming motility and promotes cellulose-dependent biofilm formation by modulating intracellular c-di-GMP levels. Functional and structural analyses revealed that the N-terminal transmembrane domains of CrdA are essential for its dimerization and enzymatic activity. Furthermore, CrdA enhances bacterial adhesion to intestinal epithelial cells and gut colonization *in vivo*, with its expression being induced upon host cell contact. Mechanistically, CrdA suppresses motility partially through the c-di-GMP effector YcgR and regulates biofilm formation in a BcsA-dependent manner. Collectively, this work elucidates a novel signaling pathway where CrdA integrates environmental cues to orchestrate a virulence-associated lifestyle switch, advancing our understanding of c-di-GMP signaling in *Cronobacter* pathogenicity and highlighting a potential target for disrupting biofilm-mediated persistence in food systems.

## Introduction

The genus *Cronobacter* consists of Gram-negative enteric pathogenic bacteria, which are widely distributed in the environment and food (Ma et al., 2024; Ye et al., 2017). To date, this genus has been clearly confirmed to comprise seven species, namely *C. malonaticus, C. sakazakii, C. condimenti, C. dublinensis, C. turicensis, C. muytjensii, and C. universalis* (Ma et al., 2024; Stephan et al., 2014). In general, *Cronbacter* can induce severe infectious diseases in humans, such as sepsis, meningitis, and necrotizing enterocolitis. It is particularly highly pathogenic to neonates and infants with compromised immune function, with the mortality rate of relevant clinical infection cases reaching 40%–60% (Joseph and Forsythe, 2012; Zeng et al., 2020).

*Cronobacter* exhibits distinct biochemical properties, including being oxidase-negative and catalase-positive, and is classified as a facultative anaerobic bacterium (Iversen et al., 2007). It can survive under extreme environmental conditions such as high temperature (Jaradat et al., 2014), extreme desiccation, and acidity (Joseph et al., 2012). Researchers hypothesize that the robust survival advantage of *Cronobacter* may rely on biofilm formation, a key strategy employed by foodborne pathogens to resist environmental stressors and achieve adaptive survival (Boles et al., 2004; Iversen et al., 2004). Biofilm is a community structure formed by the aggregation of a large bacterial population. Biofilm formation strengthens bacterial adhesion and facilitates intestinal colonization, thus enhancing pathogenicity (Boles et al., 2004; Iversen et al., 2004; O’Toole et al., 2000). Biofilms is a dynamic process that typically proceeds through five stages: “adhesion, adherence development, maturation, and dispersion” (Sauer et al., 2022). Initial bacterial adhesion and motility are critical determinants during early biofilm development. Adhesion is mediated by factors including fimbrial and non-fimbrial adhesins and exopolysaccharides (EPS) (lipopolysaccharides and cellulose) (Berne et al., 2015; Kyere et al., 2018; Liu et al., 2021), while motility is essential for efficient host contact and surface colonization (Rumbaugh and Sauer, 2020). In flagellated bacteria, motility relies on flagellar assembly and rotation, a process governed by the hierarchical FlhDC/FliA transcriptional cascade. In this pathway, FlhDC activates second-tier genes (e.g., *fliA*), and FliA subsequently induces third-tier genes (e.g., *fliC* encoding flagellin) (Hirakawa et al., 2023). Furthermore, biofilm formation is dynamically controlled by interconnected signaling pathways, primarily including quorum sensing (QS; Waters et al., 2008), cyclic-di-GMP signaling (Chen et al., 2020), and two-component system (TCS; Fernandez-Ciruelos et al., 2023).

Cyclic diguanylate is an ubiquitous bacterial second messenger that coordinately regulates key physiological processes, including the cell cycle, motility, and virulence. It governs the lifestyle transition from acute to chronic infection and reinforces biofilm integrity by stimulating the production of EPS and adhesins (Jenal et al., 2017). The intracellular levels of c-di-GMP are determined by its biosynthesis and turnover. Biosynthesis is catalyzed by DGCs with the GGDEF domain, which cyclize two GTP molecules, while degradation occurs via phosphodiesterases (PDEs) containing either EAL or HD-GYP domains, which hydrolyze the molecule to pGpG or GMP, respectively (Valentini and Filloux, 2019). Some DGC and PDEs function as “c-di-GMP sensors”, through direct interactions with effectors, facilitating downstream c-di-GMP signaling cascades, thereby enabling precise spatiotemporal regulation of bacterial physiology (Jia et al., 2025; Lindenberg et al., 2013; Richter et al., 2020).

*Cronobacter malonaticus* is a species within the genus *Cronobacter* with a relatively high clinical isolation rate, and it is closely associated with human infections (Alsonosi et al., 2015; Ma et al., 2024). Currently, researches mainly focus on stress response mechanisms, isolation and identification of drug resistance or virulence genes, and establishment of detection methods (Alvarez-Ordóñez et al., 2012; Ji et al., 2021; Ma et al., 2024; Orieskova et al., 2016). However, the specific functions and regulatory mechanisms of c-di-GMP metabolizing enzymes (CMEs) in its signaling network remain unclear. To address this gap, we systematically overexpressed all putative c-di-GMP synthase genes in *C. malonaticus* and identified a novel DGC ACETRY_02220 (named *Cronobacter* Diguanylate Cyclase A; hereafter designated as CrdA). Our data showed that as a active DGC, CrdA governs the transition between motility and sessile state of *C. malonaticus*, thus contributing to cell adhesion and intestinal colonization. This study delineates the contribution of GGDEF-domain proteins to c-di-GMP signaling in *C. malonaticus*, thereby providing a theoretical foundation for controlling its infections and developing c-di-GMP-based therapies.

## Materials and methods

### Bacterial strains, plasmids, and growth conditions

The strains and plasmids used in this study are listed in Supplementary Table 1. The strains *Escherichia coli* and *C. malonaticus* used in this study strains were incubated in LB at 37 °C. Human colorectal cancer cells (HCT116) used in this study were cultured in a 37°C incubator with 5% CO_2_. The growth medium consisted of McCoy’s 5A basal medium supplemented with 10% fetal bovine serum (FBS) and 1% triple antibiotic solution as needed.

### Construction of the overexpression plasmids

To construct overexpression plasmids, primer pairs (Supplementary Table 2) were used to amplify these target genes DNA fragments from the *C. malonaticus* NWU01 genome (accession number: CP174132.1) by polymerase chain reaction (PCR). These DNA fragments were then inserted into the *Bam*HI and *Hind*III sites of pACYCTac plasmid by seamless cloning. Finally, these overexpression plasmids were verified by primer pairs (Supplementary Table 2) and DNA sequencing.

### Construction of gene deletion mutants

The gene deletion mutants Δ*crdA*, Δ*bcsA*, Δ*csgAB*, Δ*bcsA*Δ*csgAB*, Δ*ycgR*, Δ*yajQ*, and Δ*fliC* of *C. malonaticus* were constructed using the pCVD442 suicide vector method described previously (Chen et al., 2024; Philippe et al., 2004). Plasmid construction was performed according to standard protocols with the minor modification that cloning of PCR fragment into the linearized vector was accomplished using a commercial seamless cloning and assembly kit (Vazyme). pCVD442 was linearized by PCR using the primer pair pCVD442-F and pCVD442-R, and the upstream and downstream fragments of genes to be knocked out were amplified by PCR using the primers listed in Supplementary Table 2. Primer pairs *crdA*-H1F/R and *crdA*-H2F/R were used to amplify the upstream or downstream fragments of *crdA* gene. The purified *crdA*-H1 and *crdA*-H2 homologous arms were then subjected to overlap PCR with *crdA*-H1F/*crdA*-H2R primers, resulting in a DNA fragment containing the *crdA* H1 and H2. The *crdA*-H1+H2 fragment was then inserted into the linearized pCVD442 plasmid. The sucrose-treated competent cells were transformed with the plasmid pCVD442-*crdA*-H1+H2 before plating on LB plate supplemented with carbenicillin. Following a 24 h incubation period, single-crossover recombinants were observed on the agar plates. Purified single-crossover recombinants were subsequently streaked onto LB agar plates supplemented with 10% (w/v) sucrose. The resulting double-crossover recombinants were further validated via PCR amplification with the *crdA*-F/R primer set and Sanger DNA sequencing.

### Growth assay

Overnight-grown cultures of *C. malonaticus* were diluted at a 1:100 ratio and inoculated into conical flasks containing 100 mL of LB broth, followed by an additional 14 h of incubation at 37 °C. Bacterial growth kinetics were monitored via spectrophotometry by measuring the optical density (OD) at a wavelength of 600 nm at 2 h intervals. All assays were conducted in triplicate for each sample (Li et al., 2025).

### Analysis of bacterial motility

The motility of bacteria was assessed by measuring their migration diameter on LB medium containing 0.3% (w/v) agar. Briefly, the strain to be detected was inoculated into a sterile LB medium and cultured overnight at 37 °C, then each sample was transferred to a LB soft agar plate containing 0.3% (w/v) agar and cultured at 30 °C for a further 6 h culture. Finally, colony sizes were observed and average migration diameter was calculated based on the results of three independent experiments.

### Analysis of biofilm formation

Biofilm formation was quantitatively assessed using the crystal violet staining method. Briefly, overnight bacterial cultures were diluted in LB broth to an OD_600_ of 0.3 and inoculated into sterile glass tubes, followed by static incubation at 37 °C for 24 h. After incubation, the biofilms were stained with 1% (w/v) crystal violet solution for 30 min. The inner walls of the tubes were then gently rinsed with a slow stream of distilled water to remove excess dye. Subsequently, 30% (v/v) acetic acid was added to each tube, and the samples were incubated at room temperature for 10 min to solubilize the bound dye. The absorbance at 600 nm (OD_600_) was then measured to quantify biofilm biomass.

For CLSM observation, *C. malonaticus* cultures were diluted in fresh M9 medium to an OD_600_ of approximately 0.3 and statically incubated in a 24-well plate, with a sterile coverslip placed in each well. Three biological replicates were prepared for each sample. The 24-well plate was incubated statically at 37 °C for 24 h to allow biofilm formation. After incubation, the coverslips were carefully removed to ensure that the biofilm remained intact on the glass surface. SYTO 9 fluorescent dye was then applied by adding sufficient staining solution onto each coverslip to fully cover the biofilm. The samples were incubated at room temperature in the dark for 15 min. Finally, the biofilms were visualized using confocal laser scanning microscopy.

### Analysis of EPS formation and quantification

To assess EPS production, 2 μL overnight bacterial culture was spotted onto the center of a Congo red–Coomassie brilliant blue agar plate (containing 40 μg/mL Congo red and 1 μg/mL Coomassie brilliant blue R-250). Following complete drying of the inoculum, plates were incubated at 28 °C for 5 days, and colony morphology together with color-related phenotypic changes were observed and documented with a digital camera. Colonies producing EPS appeared red and wrinkled due to the binding of Congo red to the extracellular polysaccharides.

Extraction and quantification of EPS were performed as follows: 0.5 mL overnight bacterial culture was inoculated into 4.5 mL glucose-free M9 minimal medium and statically incubated at 37 °C for 2 days. Post-incubation, the sample was centrifuged at 10,000 × *g* for 10 min to collect the supernatant, to which two volumes of absolute ethanol were added and incubated at 4 °C for 16–18 h to precipitate EPS. The sample was re-centrifuged at 10,000 × *g* for 30 min; the supernatant was aspirated and discarded. Following drying, the precipitate was resuspended in 500 μL deionized water to obtain the EPS extract. For quantification, 40 μL EPS extract was mixed with 20 μL phenol-sulfuric acid reagent, followed by rapid addition of 100 μL concentrated sulfuric acid (98%, v/v). After vigorous vortexing, the mixture was incubated at room temperature for 10 min for color development, then at 30 °C for 10–20 min to stabilize the reaction. Standards were processed identically, and absorbance was measured at OD_490_.

### Intracellular c-di-GMP quantification

Determination of intracellular c-di-GMP levels was performed as follows: Overnight bacterial cultures were subcultured in fresh medium to an OD_600_ of 0.6–0.8. Thirty milliliters of culture was collected, centrifuged at 10,000 × *g* for 5 min to harvest cells, washed twice with ice-cold PBS, and resuspended in 2 mL ice-cold PBS. The suspension was incubated in a 100 °C water bath for 5 min, immediately transferred to an ice-water bath, and subjected to ultrasonic disruption (200 W, 3 s work/5 s interval, total 10 min). Post-disruption, samples were centrifuged at 10,000 × *g* for 5 min to collect lysates; pellets were resuspended, and the disruption-centrifugation cycle was repeated three times. Pooled lysates were used for c-di-GMP quantification via a c-di-GMP ELISA kit (MSKBIO) on a BioTek microplate reader, following the manufacturer’s instructions.

### Adhesion assays

HCT116 human colon cancer cells were maintained in McCoy’s 5A medium supplemented with 10% FBS and 1% penicillin-streptomycin-gentamicin solution (Sangon Biotech, Shanghai, China) at 37 °C with 5% CO_2_. Cells were seeded into 24-well plates. Bacteria grown to OD_600_≈0.8 were washed with PBS and added to the wells at a multiplicity of infection MOI = 100. After a 1.5 h incubation at 37 °C and 5% CO_2_ to allow adhesion, non-adherent bacteria were removed by three PBS washes. Adherent cells were detached by treating with 300 μL trypsin for 3-5 min at 37 °C, collected by pipetting, and transferred into 1.5 mL microcentrifuge tubes. Cells were centrifuged at 12,000 rpm for 2 min, resuspended in 1 mL of PBS, serially diluted, and plated onto LB agar. Plates were incubated overnight at 37 °C, and colony-forming units (CFUs) were enumerated the following day.

Adhesion rate was assessed by the formula shown below:

Adhesion rate (%) = $\frac{{\text{A}}_{\text{t}}}{{\text{A}}_{0}}$ 100 (1)

A_*t:*_ Adherent *Cronobacter*; A_0_: Initial number of *Cronobacter*.

### Bacterial two-hybrid experiments

To test for specific protein–protein interactions, a bacterial two-hybrid system was used (Karimova et al., 1998). Plasmids containing pUT18 and pKNT25 were constructed to generate fusion proteins. These plasmids were co-transformed into *BTH101* competent cells, and the cells were recovered for 1 h before being plated on LB agar plates containing both kanamycin and ampicillin. After overnight incubation at 37 °C, single colonies were selected for PCR validation to confirm plasmid insertion. Correctly identified colonies were cultured and then plated onto LB-X-gal solid medium containing 40 μg/mL X-gal and 0.5 mmol/L IPTG. The plates were incubated at 30 °C for 72 h. Interaction was assessed by observing the color change of the LB-X-gal plates. Blue colonies indicated a positive interaction, while non-colored colonies indicated no interaction.

### Animal experiments

All animal experiments were performed in accordance with the guidelines for the care and use of laboratory animals established by Northwest University and relevant national regulations. Bacteria were cultured overnight in LB broth, subcultured into fresh LB medium, and incubated at 37 °C with shaking until the logarithmic growth phase. After centrifugation, bacterial pellets were resuspended in 1× PBS to adjust the concentration to 1 × 10^9^CFU/mL. C57BL/6J mice (Biocytogen, both sexes, 10–15 days old) were inoculated by oral gavage with 15 μL of bacterial suspension or 15 μL of 1× PBS as a control. Infection was monitored for 24 h, and no mortality was observed during this period. At the end of the infection period, mice were anesthetized via intraperitoneal injection of 1% pentobarbital sodium at a dose of 50 mg/kg body weight. Following complete anesthesia (disappearance of corneal reflex), animals were fixed in a supine position on a dissection table. Intestinal tissues were harvested and homogenized. Bacterial loads were determined by serial dilution followed by the agar plate spread-plate method.

### Animal ethics statement

All animal experiments were performed in strict accordance with the Guidelines for the Care and Use of Laboratory Animals of the Animal Ethics Committee of Northwest University. Every effort was made to minimize animal suffering and reduce the number of animals used.

### Quantitative real-time PCR verification

Several candidate genes with differential expression were randomly selected for quantitative real-time PCR (qRT-PCR) validation, with gene-specific primers designed via Beacon Designer 8 software (Supplementary Table 2). For RNA preparation, overnight cultures of *C. malonaticus* were sub-cultured at a 1:50 dilution into fresh LB medium, and bacterial cells were harvested at log phase (OD_600_ = 0.6–0.8). Total RNA was isolated using the RNAprep pure Bacteria Kit (TIANGEN), and subsequently reverse-transcribed into complementary DNA (cDNA) with a reverse transcriptase kit (TransGen). qRT-PCR amplification was carried out using SuperReal PreMix Plus (SYBR Green) reagent (TIANGEN), with cycling conditions set as follows: initial denaturation at 95 °C for 15 min, followed by 40 cycles of denaturation at 95 °C for 10 s, annealing at 56 °C for 20 s, and extension at 72 °C for 20 s. Amplicon specificity was verified via melting curve analysis, with the gyrB gene serving as the endogenous reference. All qRT-PCR assays were performed with at least three independent replicates per sample. Relative gene expression levels were calculated using the 2^–Δ Δ *Ct*^ method (Li et al., 2025).

### Statistical analysis

Unless otherwise indicated, data are presented as mean ± standard deviation (SD) of three independent biological experiments (*n* = 3). Statistical analyses were performed in GraphPad Prism 9.5 using One-way/Two-way ANOVA. Statistical significance was defined as *P* < 0.05, and significant differences between groups were indicated by a single asterisk (*) in the figures.

## Results

### Systematic analysis of genes encoding GGDEF domain-containing protein in *C. malonaticus*

Analysis of the genome of *C. malonaticus* NWU01 revealed 13 genes encoding predicted c-di-GMP synthase with GGDEF domain (Blum et al., 2025). BLAST analysis revealed that the several c-di-GMP synthases from *C. malonaticus* share significant sequence homology with those from *E. coli* (Figure 1a). Multiple modular domains are associated with the GGDEF domain (Figure 1b), including representative signal-sensing or function-regulating domains such as the transmembrane (TM), Per-ARNT-Sim (PAS), GAF, and PDC. Among these domains, TM was the most prevalent identified in our analysis, indicating the membrane-binding property of 10 of the 13 putative DGCs.

**FIGURE 1:**
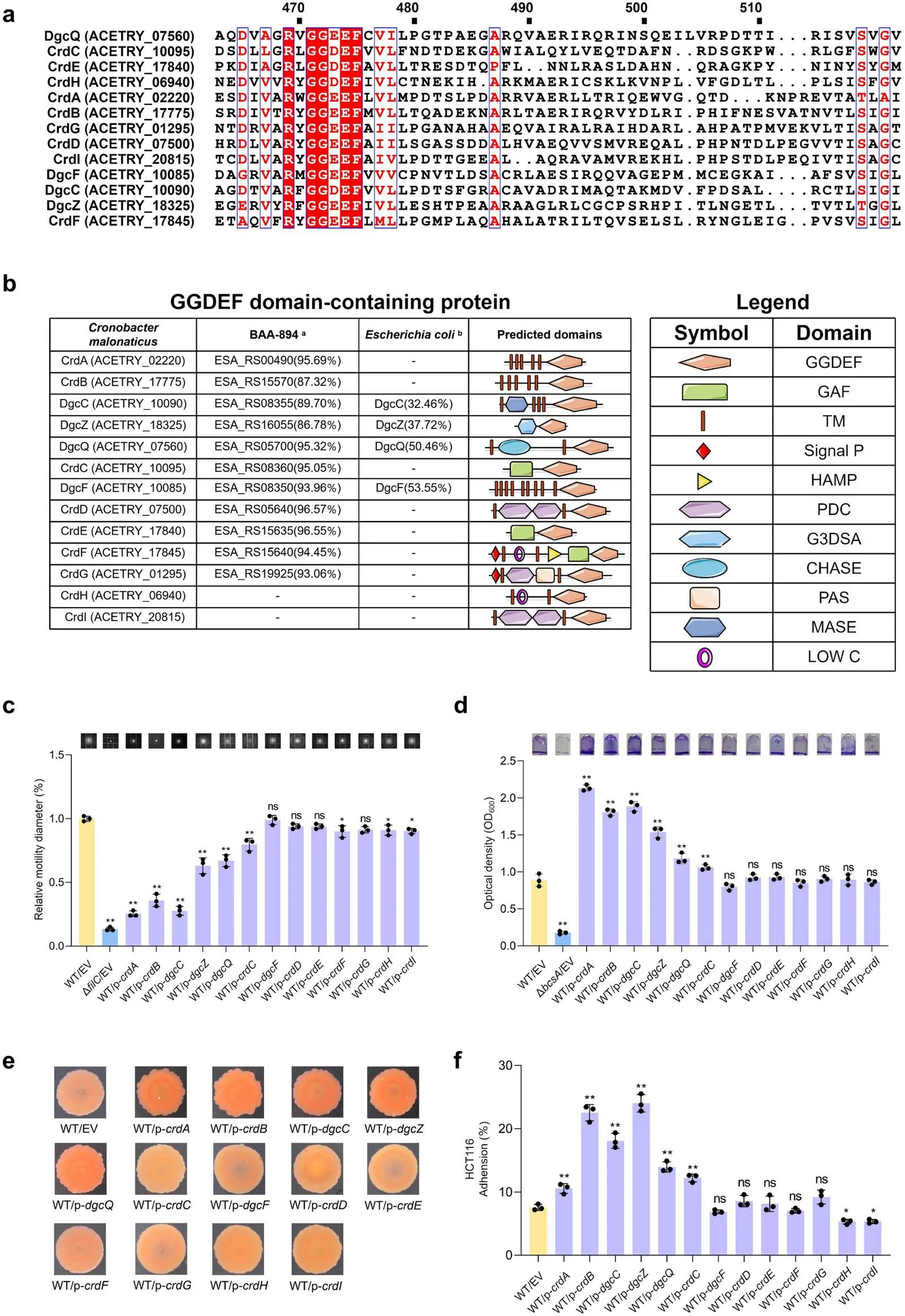
Systematic analysis of genes encoding GGDEF domain-containing proteins in *C. malonaticus*. **(a)** Conserved domains in the amino acid sequences of GGDEF domain-containing proteins. **(b)** Schematic diagram of the secondary structure of GGDEF domain-containing proteins. **(c)** Motility assay of the strains overexpressing GGDEF domain-encoding genes. Growth phenotypes of the strains following 6 h of incubation on motility agar at 30 °C (Top) and quantification of colony migration diameter and statistical analysis (Bottom). **(d)** Biofilm formation of the strains overexpressing GGDEF domain-encoding genes. Visualization of biofilm formation by the indicated strains via crystal violet staining (Top) and quantification through OD_600_ measurement (Bottom). **(e)** Cellulose production assay of the strains overexpressing GGDEF domain-encoding genes. Colony morphology of strains after 5 days of growth on Congo red agar plates at 28 °C. **(f)** Cell adhesion assay of strains overexpressing GGDEF domain-encoding genes. HCT116 cells infected with the strains for 1.5 h were processed, followed by serial dilution, plating, and colony counting to calculate the adhesion rate. Wild-type (WT) refers to the wild-type *C. malonaticus* strain NWU01. Empty vector (EV) denotes the empty pACYCTac plasmid transformed into the WT strain. p-*crdA* and other plasmids represent overexpression constructs (e.g., pACYCTac-*crdA*) harboring additional GGDEF domain-encoding genes, which were introduced into the WT strain. Error bars means SD, *n* = 3. **P < 0.01 and *P < 0.05.

To systematically screen the putative DGCs that regulate bacterial swimming motility, a library of overexpression vectors targeting each gene encoding the putative DGC was constructed. Of the 13 overexpression strains, 6 showed a decrease in swimming motility compared to the wild type. The motility of three strains (CrdA, DgcC, and CrdB) was significantly reduced. Three strains (DgcZ, and DgcQ, CrdC) showed ∼80% of wild-type levels of motility (Figure 1c).

Biofilm formation results showed that strains overexpressing *crdA*, *dgcC*, *crdB*, or *dgcZ* were all hyperbiofilm producers, ranging from 160% to 200% of wild-type strain levels (Figure 1d). Cellulose is an extracellular matrix component in *C. sakazakii* biofilms (Grimm et al., 2008). Notably, putative gene clusters for cellulose and curli fimbriae were both identified in *C. malonaticus* (Joseph et al., 2012). Macrocolony test with the matrix dye Congo red showed that a strain that produces cellulose only (Δ*csgBA*) showed no significant difference with the wild type, whereas curli-only producer (Δ*bcsA*) and double mutant that eliminate both curli and cellulose production generates unstructured and white colonies not stained with the matrix dye CR (Supplementary Figure 1). This result indicated that cellulose is the primary component of extracellular polymeric matric in *C. malonaticus*. As shown in Figure 1e, 5 (CrdA, DgcC, CrdB, DgcZ, and DgcQ) of the 13 strains showed dark red and increased wrinkly macrocolony morphology compared to wild type, indicating more cellulose production.

Adhesion to the host cell is an initial and crucial step in the pathogenesis of foodborne pathogens (Fan et al., 2023; Rehman et al., 2019). The roles of the DGCs in intestinal cell adhesion were assessed in *C. malonaticus*. Among the 13 overexpression strains, 6 strains exhibited increased adhesion to HCT116 cells. Particularly, 3 strains (CrdB, DgcC, and DgcZ) showed significant increases in adhesion, with ∼240%–300% of wild-type levels (Figure 1f). The other 3 strains (CrdA, DgcQ and CrdC) showed ∼150%–200% of wild-type levels of adhesion (Figure 1f).

### CrdA reciprocally regulate motility and biofilm formation in *C. malonaticus*

The results above showed that CrdA significantly regulates bacterial motility, biofilm formation, and bacterial adhesion. To our knowledge, CrdA and its close homologs have not been functionally or mechanistically characterized, which motivated our selection of CrdA for detailed analysis. Therefore, a Δ*crdA* mutant (Δ*crdA/*EV) was constructed to pursue the role of CrdA in the regulation of motility and biofilm formation. Deletion of *crdA* showed no impact on bacterial growth (Supplementary Figure 2). As shown in Figure 2a, the Δ*crdA*/EV mutant exhibited stronger motility (115% of the wild type), which was suppressed by complementation of *crdA*. This results showed that CrdA repress swimming motility of *C. malonaticus*.

**FIGURE 2:**
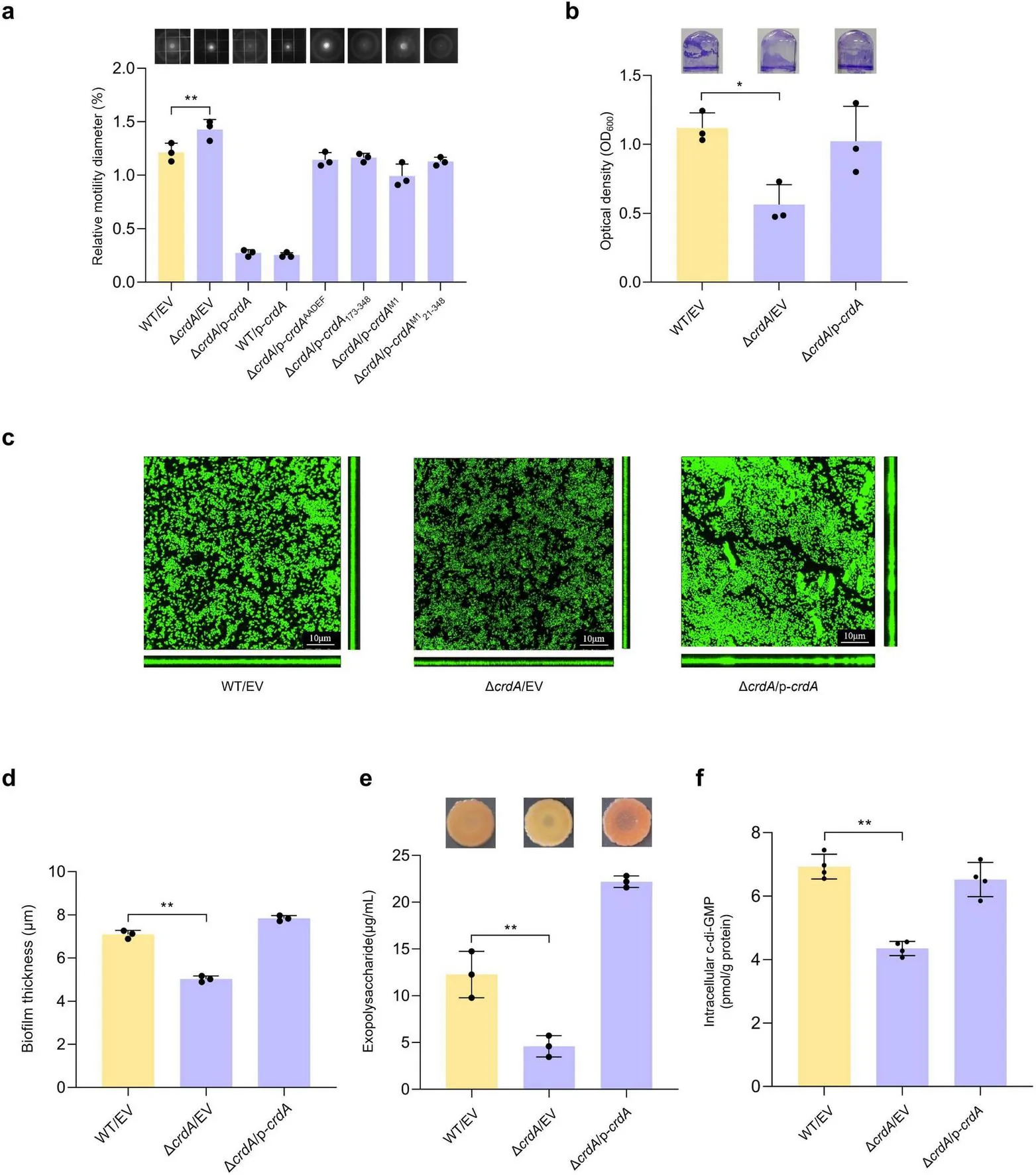
CrdA reciprocally regulates motility and biofilm formation in *C. malonaticus*. **(a)** CrdA inhibits the motility of *C. malonaticus*. Growth phenotypes of the WT/EV, Δ*crdA*/EV, and Δ*crdA*/p-*crdA* strains following 6 h of incubation on motility agar at 30 °C (Top) and quantification of colony migration diameter and statistical analysis (Bottom). **(b)** CrdA promotes biofilm formation in *C. malonaticus*. Biofilm formation was stained by crystal violet (Top) and quantification through OD_600_ measurement (Bottom). **(c)** Biofilm formation in the WT/EV, Δ*crdA*/EV, and Δ*crdA*/p-*crdA* strains via CLSM. **(d)** Quantification of biofilm thickness of the WT/EV, Δ*crdA*/EV, and Δ*crdA*/p-*crdA* strains based on CLSM images. **(e)** Quantification of EPS production in the WT/EV, Δ*crdA*/EV, and Δ*crdA*/p-*crdA* strains. **(f)** Intracelluar c-di-GMP in the WT/EV, Δ*crdA*/EV, and Δ*crdA*/p-*crdA* strains were determined by EliSA Kit. WT refers to the wild-type *C. malonaticus* strain NWU01. EV denotes the empty pACYCTac plasmid transformed into the WT strain. p-*crdA* represent the overexpression construct pACYCTac-*crdA*. Error bars means SD, *n* = 3. **P < 0.01 and *P < 0.05.

Then, the biofilm formation of Δ*crdA* mutant was determined. As illustrated in Figure 2b, the deletion of *crdA* reduced biofilm formation relative to the wild-type strain. In contrast, complementation of *crdA* gene significantly enhanced biofilm formation, with its biofilm biomass being approximately threefold higher than that of the wild-type strain. Additionally, the biofilm thickness of the WT/EV, Δ*crdA*/EV, and Δ*crdA*/p-*crdA* strains was examined via CLSM. As presented in Figures 2c,d, the average biofilm thickness of the WT/EV strain was approximately 7.12 μm, thicker than that of the Δ*crdA* mutant (5.17 μm). The Δ*crdA* mutant with p-*crdA* plasmid exhibited an average biofilm thickness of ∼7.97 μm, comparable to that of the wild-type strain. Furthermore, colony morphology and quantitative analysis of EPS showed that deletion of *crdA* significantly inhibited bacterial EPS production (Figure 2e). Collectively, these findings demonstrate that CrdA positively regulates the biofilm formation of *C. malonaticus.*

### The DGC activity is essential for CrdA to regulate biofilm and motility

CrdA was a putative DGC with active GGDEF domain (Figure 1b). To confirm the activity of DGC CrdA, we measured the intracellular c-di-GMP level in the WT/EV, Δ*crdA*/EV, and Δ*crdA*/p-*crdA* strains via an ELISA kit. Consistent with the predicted DGC function of CrdA, the Δ*crdA* mutant produced significantly lower c-di-GMP than the WT strain (Figure 2f). To further elucidate that the DGC activity of CrdA is indispensable for its regulatory role in bacterial motility and biofilm formation, a point-mutated plasmid p-*crdA^G266A G267A^* was constructed and introduced into Δ*crdA* mutant, ultimately yielding the point-mutated strain Δ*crdA/*p-*crdA^AADEF^*. As shown in Figure 2a, introduction of p-*crdA^AADEF^* in Δ*crdA* mutant did not repress bacterial motility. On Congo red agar plates, the Δ*crdA/*p-*crdA^AADEF^* strain exhibited pink, smooth colony morphology (Supplementary Figure 3), which was largely consistent with that of Δ*crdA*. These results indicated that mutation of the CrdA active site abolishes its DGC activity.

### The N-terminal domain of CrdA is critical for its dimerization and DGC activity

As is well established, GGDEF domain-containing proteins are typically activated via dimerization, which enables them to exert their DGC activity to catalyze the synthesis of c-di-GMP (Chen et al., 2021; De et al., 2008; Wassmann et al., 2007). InterPro database revealed that residues 24–172 of CrdA contain five transmembrane domains (Figure 1b). To test the potential of CrdA to form dimer, the dimeric interface of two CrdA molecules bound to two GTP molecules was predicted using AlphaFold3. As shown in Figure 3a, three pairs of amino acid residues (Y135–E95, Y135–N153, and R138–N153) at the N-terminus of CrdA stabilize the homodimer via hydrogen bonding. Additionally, C-terminal residues interact with N-terminal residues (D5, Q8, L9) of the opposing monomer (Supplementary Table 3). This analysis suggested that the N-terminal TM domains are important for CrdA dimerization. To test this, a bacterial two-hybrid system was employed. The bait plasmids pUT18-*crdA*, pUT18-*crdA*^173–348^ and prey plasmids pKNT25-*crdA*, pKNT25-*crdA*^173–348^ (truncated CrdA without the N-terminal transmembrane domain coding sequence) were constructed. Meanwhile, we generated another two-hybrid plasmids pUT18-*crdA*^*M*1^(*crdA*^*E*95*AY*135*AR*138*AN*153*A*^) and pKNT25-*crdA*^*M*1^(*crdA*^*E*95*AY*135*AR*138*AN*153*A*^), which harbor point mutations in three amino acids pairs that form stable hydrogen bonds at the N-terminus. As depicted in Figure 3b, blue colonies were observed for CrdA, indicating that the protein undergoes dimerization. In contrast, the CrdA^173–348^ and CrdA^*M*1^ showed no color change relative to the negative control, suggesting that the N-terminal TM domains mediate dimerization of CrdA. Additionally, mutation of the key amino acids located at the dimer interface of CrdA predicted by AlphaFold3 significantly impact the self-interaction of CrdA. These results showed that the N-terminal was critical for CrdA dimerization.

**FIGURE 3:**
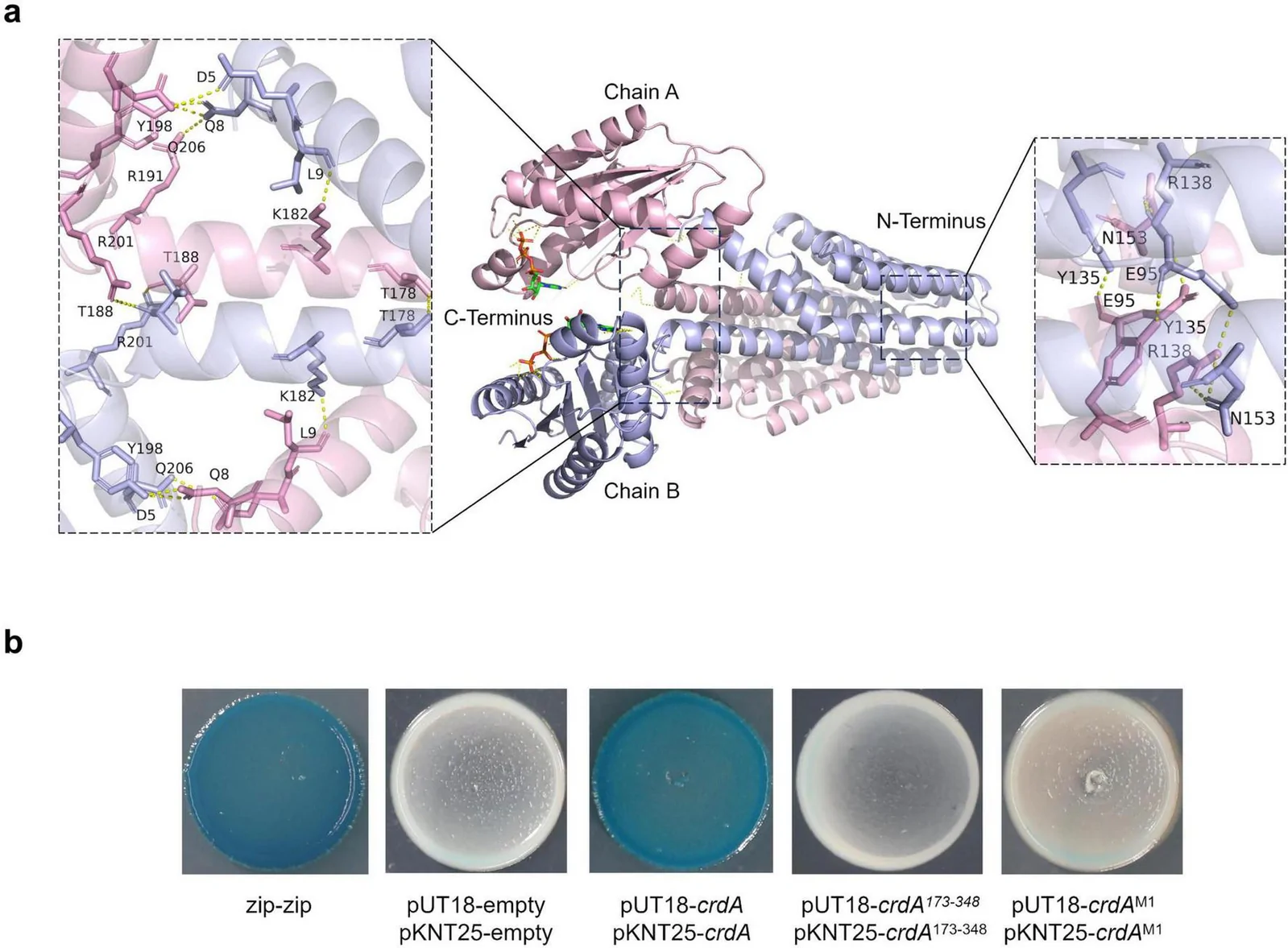
The N-terminal domain of CrdA is critical for its dimerization and DGC activity. **(a)** Overall fold of the CrdA dimer-GTP complex. The two CrdA chains are displayed as cartoon representations in light purple and pink, respectively. GTP is shown as a colored stick model. **(b)** Self-interaction of CrdA, CrdA^173– 348^ and CrdA^M1^ were identified via bacterial two-hybrid assay. The positive control (Zip-Zip) yielded blue colonies, while the negative control produced white colonies.

To investigate whether the N-terminal domains were essential for CrdA activity, phenotypic experiments were performed. As shown in Figure 2a, overexpression of truncated *crdA*^173–348^ gene showed no impact on the swimming motility of Δ*crdA* mutant, suggesting that the N-terminal TM domains were critical for CrdA activity. Additionally, the key amino acids predicted to be located at the dimer interface of CrdA were site-directed. Compared to wild-type *crdA*, overexpression of *crdA*^*M*1^(*crdA^E95AY135AR138AN153A^*) in Δ*crdA* mutant partially relieved the repression of swimming motility. Further deletion of the first 20 amino acids (CrdA^*M*1^_21–348_) completely abolished the regulatory role of CrdA in motility (Figure 2a). Similarly, we observed that the Δ*crdA*/p-*crdA*^173–348^, Δ*crdA*/p-*crdA*^*M*1^ and Δ*crdA*/p-*crdA*^*M*1^_21–348_ strains exhibited pink, smooth colonies on Congo red agar plates (Supplementary Figure 3). These results demonstrated that the N-terminal domain was essential for CrdA DGC activity by stabilizing the dimer formation.

### CrdA regulates bacterial motility at transcriptional and post-transcriptional levels

As confirmed in previous studies, c-di-GMP exerts functional regulation via its downstream effectors (Jia et al., 2025). YcgR, an effector containing a PilZ domain, is an inhibitor of bacterial motility (Boehm et al., 2010). To investigate whether CrdA regulates bacterial motility via YcgR, we constructed a *ycgR* knockout strain. As depicted in Figure 4a, deletion of the *ycgR* gene partially relieved the strong repression of CrdA on swimming motility. Furthermore, a bacterial two-hybrid assay was performed to test direct interaction between CrdA and YcgR. However, no direct interaction was observed between CrdA and YcgR (Supplementary Figure 4). Morever, another PilZ domain-containing effector, YajQ, serves as a motility regulator in *Xanthomonas campestris* (An et al., 2014) and *Salmonella* (Dai et al., 2024). However, deletion of the *yajQ* gene did not affect CrdA-mediated repression of bacterial motility (Supplementary Figure 5). These results suggest that CrdA partially inhibits motility via the c-di-GMP effector YcgR, which acts downstream of c-di-GMP to inhibit flagellar motility post-transcriptionally.

**FIGURE 4:**
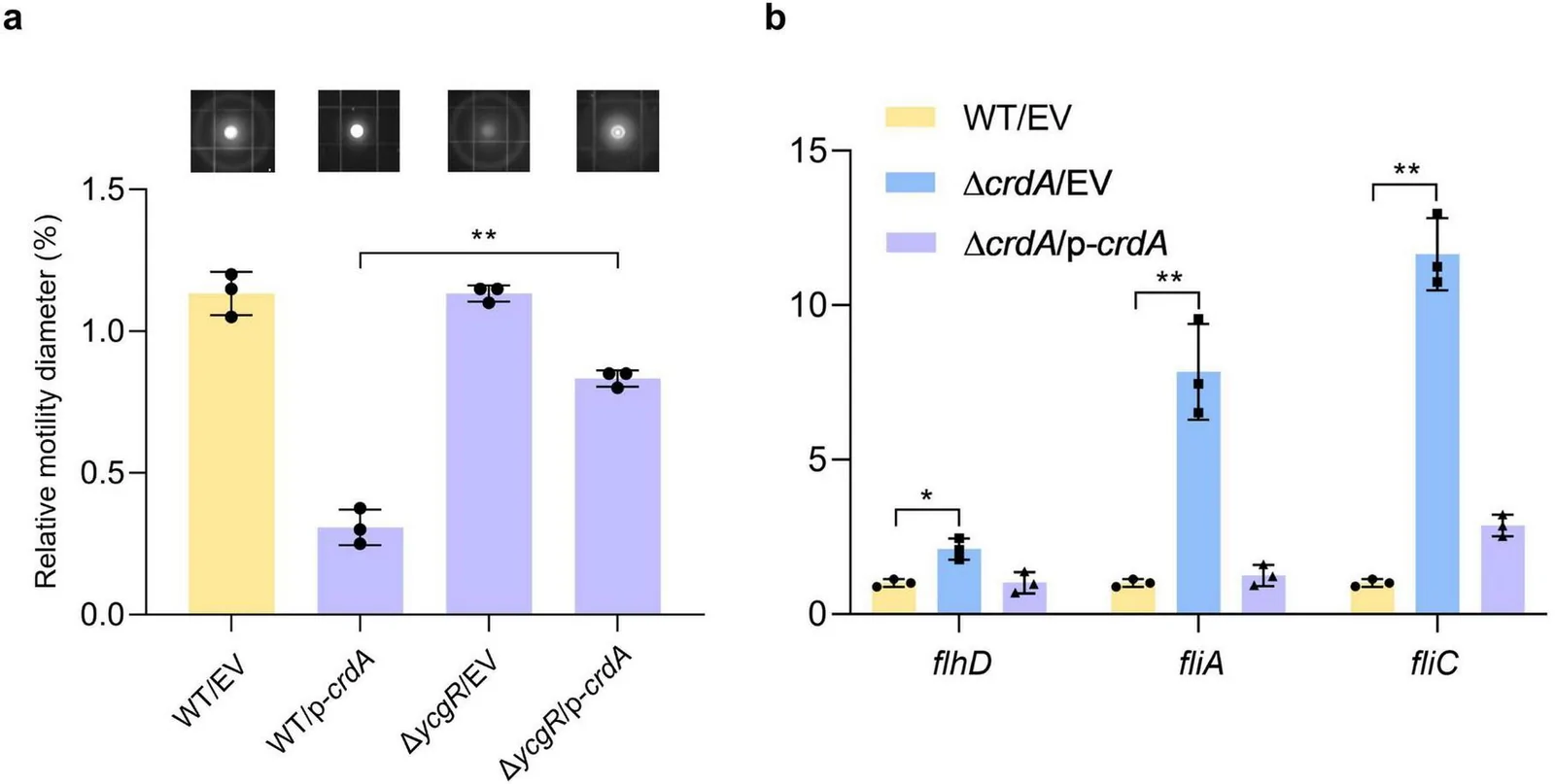
CrdA regulates bacterial motility at transcriptional and post-transcriptional levels. **(a)** CrdA regulates bacterial motility partially through a YcgR-dependent pathway. Growth phenotypes of the strains following 6 h of incubation on motility agar at 30 °C (Top) and quantification of colony migration diameter and statistical analysis (Bottom). **(b)** Relative expression levels of *flhD*, *fliA*, and *fliC* in the WT/EV, Δ*crdA*/EV, and Δ*crdA*/p-*crdA* strains were determined by qRT-PCR. WT refers to the wild-type *C. malonaticus* strain NWU01. EV denotes the empty pACYCTac plasmid transformed into the WT strain. p-*crdA* represent the overexpression construct pACYCTac-*crdA*. Error bars means SD, *n* = 3. **P < 0.01 and *P < 0.05.

Additionally, the regulatory role of CrdA on the expression of flagellar biosynthesis genes was also tested. We performed qRT-PCR to quantify the expression of *flhD*, *fliA*, and *fliC* genes in the Δ*crdA* mutant. As shown in Figure 4b, the transcription levels of *flhD*, *fliA*, and *fliC* were elevated in the Δ*crdA* strain, indicating that CrdA may regulates the expression of flagellar genes at the transcriptional level. Collectively, these data indicated that CrdA also regulates bacterial motility at transcriptional level in *C. malonaticus*.

### CrdA contributes to EPS production and biofilm formation in a BcsA-dependent manner

To assess the role of CrdA in EPS biosynthesis, macrocolony morphology test was performed. WT/EV colonies exhibited a pink, wrinkled appearance, while the Δ*crdA*/EV mutant displayed a smooth, pale pink morphology (Supplementary Figure 3). In contrast, the complemented strain formed dry, red, and highly wrinkled colonies (Supplementary Figure 3), indicating that CrdA enhanced EPS production. This result suggested that deletion of *crdA* impairs EPS synthesis, consistent with its reduced biofilm formation capacity. Given that cellulose is a known EPS component in *C. sakazakii*, we next examined whether the effects of CrdA on EPS and biofilm are dependent on cellulose biosynthesis in our strain. Therefore, we constructed a cellulose synthase subunit knockout strain (Δ*bcsA*) and introduced the p-*crdA* plasmid into this background. As shown in Figure 5a, both the Δ*bcsA* and Δ*bcsA*/p-*crdA* strains formed white, smooth colonies, and failed to produce detectable EPS production, even when *crdA was* overexpressed. Subsequently, we assessed the biofilm formation capacity of the Δ*bcsA* and Δ*bcsA*/p-*crdA* strains. Consistently, these two strains displayed relatively weak biofilm formation (Figure 5b), indicating that *crdA* overexpression fails to enhance biofilm formation in Δ*bcsA* mutant. These results suggest that CrdA influences biofilm formation in a BcsA-dependent manner.

**FIGURE 5:**
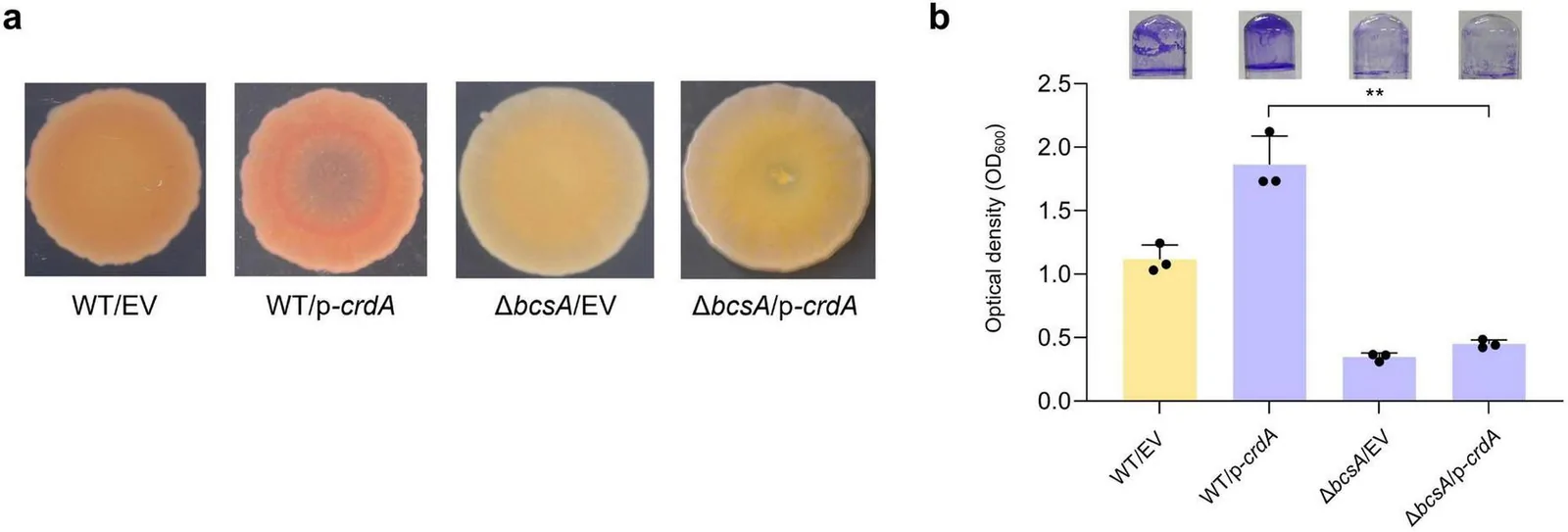
CrdA regulates EPS production and biofilm formation through BcsA. **(a)** Cellulose production assay of the strains overexpressing GGDEF domain-encoding genes. Colony morphology of strains after 5 days of growth on Congo red agar plates at 28 °C. **(b)** Biofilm formation assay of the Δ*bcsA* and Δ*bcsA*/p-*crdA* strains. Visualization of biofilm formation by the indicated strains via crystal violet staining (Top) and quantification through OD_600_ measurement (Bottom). WT refers to the wild-type *C. malonaticus* strain NWU01. EV denotes the empty pACYCTac plasmid transformed into the WT strain. p-*crdA* represent the overexpression construct pACYCTac-*crdA*. Error bars means SD, *n* = 3. **P < 0.01.

To further determine whether CrdA modulates the function of the cellulose synthase complex through direct binding interactions, we employed a bacterial two-hybrid system to test interactions between CrdA and the cellulose biosynthesis components BcsA and BcsB. However, no detectable interaction signals were observed between CrdA and either BcsA or BcsB (Supplementary Figure 6), indicating the absence of direct physical binding. This result suggested that CrdA does not regulate cellulose production by directly binding to the Bcs complex.

### CrdA enhances the cell adhesion of *C. malonaticus*

Bacterial adhesion is critical for bacteria to achieve colonization, infection, and environmental adaptation (Carniello et al., 2018). To determine the role of CrdA in cell adhesion of *C. malonaticus*, we performed cell adhesion assay. As shown in Figure 6a, the adhesion rate of the Δ*crdA* mutant was lower than that of the wild-type strain, reaching approximately 43% of the wild-type level. In contrast, the complemented strain exhibited adhesion capacity comparable to that of the wild-type. Subsequently, the expression of the *crdA* gene in the wild-type strain was quantified during its adhesion to HCT116 cells. As shown in Figure 6b, adhesion to host cells specifically induced the expression of *crdA*, whereas the expression of the control gene *crdI* was not altered. These results indicate that CrdA positively contributes to the adhesion of *C. malonaticus* to intestinal cells.

**FIGURE 6:**
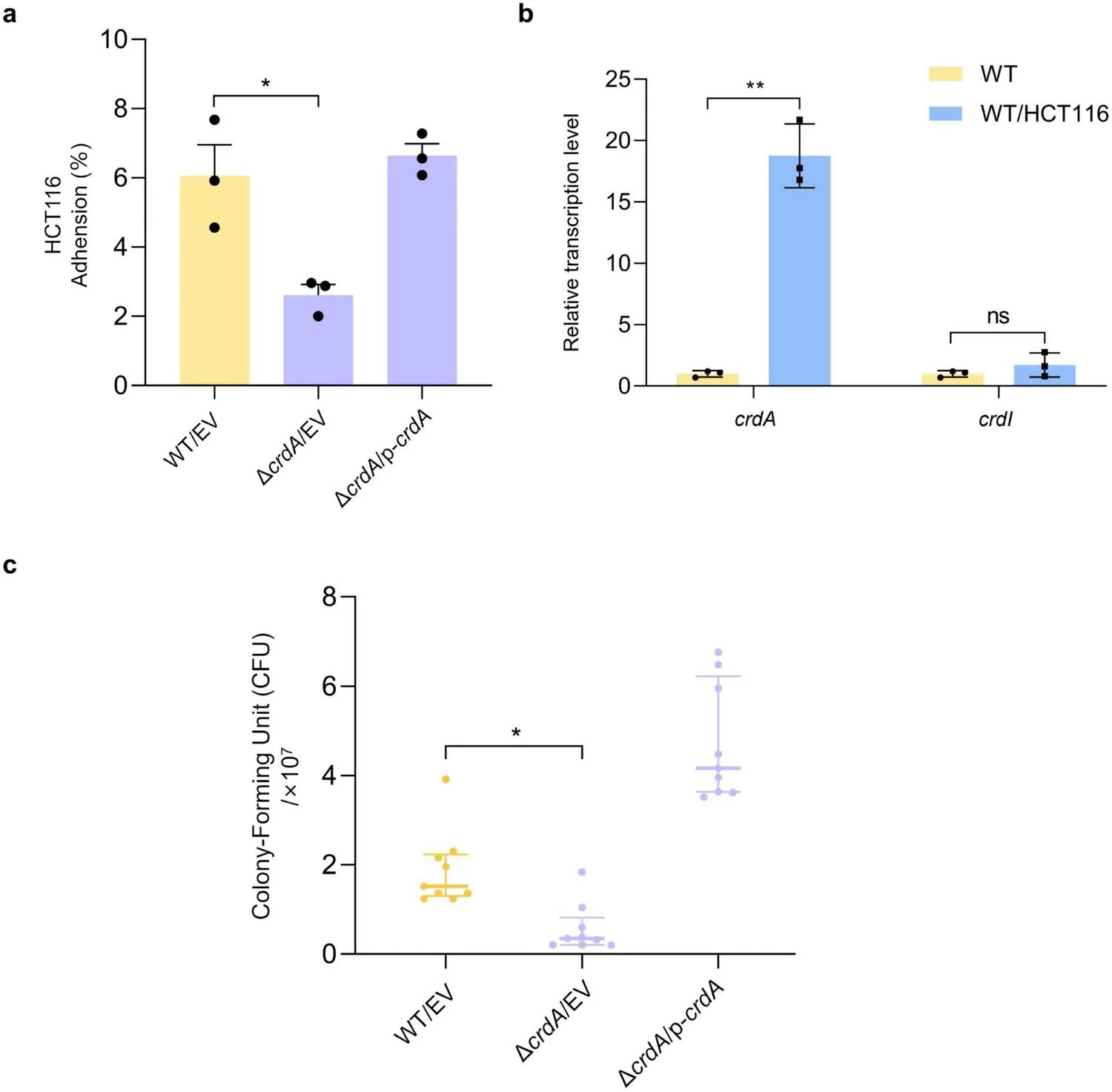
CrdA enhances adhesion and intestinal colonization of *C. malonaticus*. **(a)** Adhesion of *C. malonaticus* strains to HCT116 cells. HCT116 cells were infected with WT/EV, Δ*crdA*/EV, or Δ*crdA*/p-*crdA*. **(b)** Detection of *crdA* expression levels in wild-type strains following infection of HCT116 cells. *crdI* expression showed no significant differences before and after HCT116 cell infection, serving as a negative control. **(c)** CrdA enhances intestinal colonization of *C. malonaticus* in neonatal rats. Neonatal rats (10–15 days old) were orally infected with WT/EV, Δ*crdA*/EV, or complemented Δ*crdA*/p-*crdA* strains. At the indicated time point post-infection, bacterial colonization in the intestinal tract was quantified by plating homogenized intestinal samples. Error bars means SD, *n* = 3. **P < 0.01 and *P < 0.05.

### CrdA affects the gut colonization ability of *C. malonaticus in vivo*

Efficient adhesion to intestinal epithelia is a prerequisite for successful colonization and subsequent infection (Jans et al., 2023; Jans et al., 2024). To investigate the role of CrdA in intestinal colonization, an *in vivo* infection model was established using neonatal mouse. As shown in Figure 6c, the Δ*crdA* mutant exhibited significantly reduced intestinal colonization compared to the wild-type group. Importantly, the complemented strain restored colonization ability to levels comparable to those of the wild-type, suggesting that CrdA contributes to intestinal colonization of *C. malonaticus*.

### CrdA regulates the transcription of virulence genes in *C. malonaticus*

Previous studies have shown that the expression of environmental tolerance factors and virulence genes in *C. sakazakii* is regulated by several regulatory factors (Jin et al., 2024; Maerani et al., 2020; Sun et al., 2022). To test whether CrdA regulates these gene in *C. malonaticus*, qRT-PCR test was performed in the Δ*crdA* mutant. Results showed that transcription levels of genes associated with outer membrane proteins (*ompA*, *ompC*, *ompX*, *ompR*) were significantly upregulated in the Δ*crdA* mutant (Figure 7a). Transcription of host invasion–related genes (*urvY*, *luxR*, *wzx*) was significantly reduced (Figure 7b). Genes involved in antigen synthesis (*flgJ*, *sodA*, *lpxB*), *flgJ* and *sodA* were significantly upregulated, while *lpxB* was markedly downregulated (Figure 7c). Within the stress response regulatory genes (*rpoS*, *rpoN*, *fur*, *cpxR*, *groES*, *osmY*, *mfla*), *rpoS*, *groES*, and *cpxR* showed significantly elevated expression (Figure 7d). Furthermore, complementation with *crdA* restored the transcription levels of these genes, supporting its regulatory role in modulating virulence-associated gene expression in *C. malonaticus*.

**FIGURE 7:**
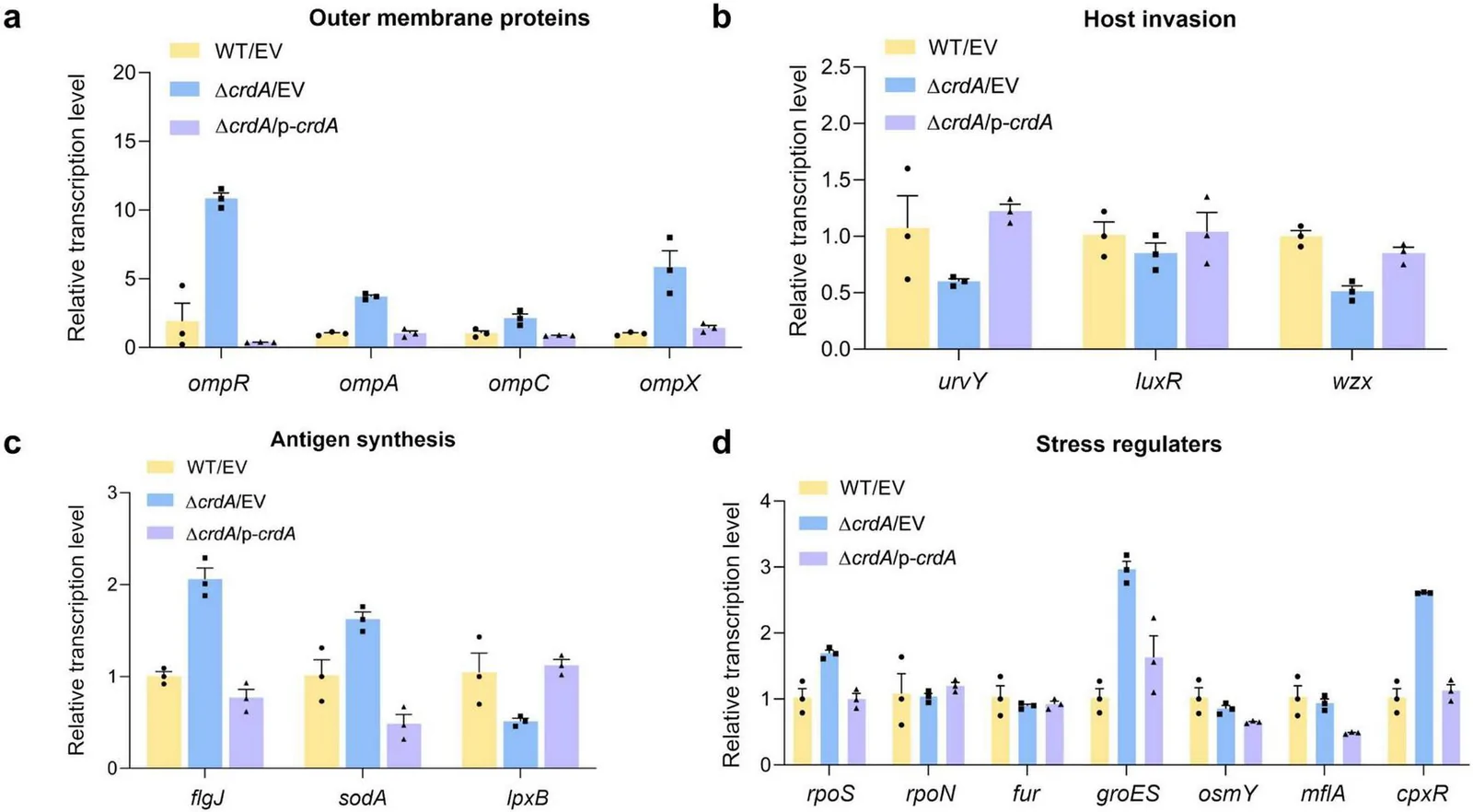
qRT-PCR analysis of the expression of virulence genes in the WT/EV, Δ*crdA*/EV, and Δ*crdA*/p-*crdA* strains. **(a)** Outer membrane proteins (*ompA*, *ompC*, *ompX*, *ompR*). **(b)** Host invasion–related genes (*urvY*, *luxR*, *wzx*). **(c)** Antigen synthesis–related genes (*flgJ*, *sodA*, *lpxB*). **(d)** Stress response regulatory genes (*rpoS*, *rpoN*, *fur*, *cpxR*, *groES*, *osmY*, *mfla*).

## Discussion

*Cronobacter malonaticus* is a rare but life-threatening foodborne pathogen of significant importance, and the risks it poses to public health have garnered considerable attention (Ling et al., 2022). Meanwhile, c-di-GMP, a second messenger widely distributed across bacterial species, regulates various physiological and metabolic activities in bacteria (Kreiling and Thormann, 2023). In this study, we determined the effect of 13 putative DGCs on c-di-GMP-mediated phenotypes in *C. malonaticus* and found that 5 (CrdA, DgcC, CrdB, DgcZ, and DgcQ) of the 13 putative DGCs regulate multiple phenotypes. The functions of DgcC, DgcQ and DgcZ have been investigated in *E. coli*. Physical proximity between cellulose synthase and localized c-di-GMP pool is established through direct interactions of DgcC and PDE PdeK with cellulose synthase subunit BcsB and with each other (Richter et al., 2020). Dual allosteric regulation of the diguanylate cyclase DgcQ by UTP and N-carbamoyl-aspartate directly links cellulose biosynthesis to the pyrimidine salvage pathway (Rossi et al., 2017). DgcZ is the main DGC regulating EPS production under the control of complex transcriptional and post-transcriptional regulations (Xu et al., 2019). However, CrdA and CrdB are two novel DGCs without known function previously. This study focused on the function of CrdA. And the DGC CrdB was also under investigation in our lab.

Diguanylate cyclase catalyzes the condensation of two identical substrate molecules of GTP to form the twofold symmetric c-di-GMP molecule. Since the GGDEF domain binds only one substrate, DGC activity relies on an oligomerization event that brings two GGDEF domains into close proximity. Oligomerization is involved in the modulation of WspR activity through the switch from an active to a product-inhibited dimer via tetrameric assembly (De et al., 2008). Additionally, active PleD holds a dimer conformation, which is modulated by the phosphorylation of its active aspartate residue (Wassmann et al., 2007). Similarly, we previously found that DGC SiaD is activated by partner protein SiaC through the SiaC-SiaD interaction-mediated dimer formation (Chen et al., 2021). Here, our experiment data as well as AlphaFold3 analysis demonstrated that the N-terminus of CrdA was essential for its dimer formation and enzyme activity. The N-terminus of CrdA stabilizes its dimer formation through two ways, that is, the N-terminal TM–TM interactions (Y135–E95, Y135–N153, and R138–N153) and the N terminus-C terminus interactions (D5–R191/Y198, Q8–Q206, L9–K182) (Supplementary Table 3). Similarly, the N-terminal TM domains of SadC in *P. aeruginosa* was essential for its regulation role in motility and EPS production, although the remaining truncated SadC was still able to synthesize c-di-GMP. The N-terminus of CrdA may facilitate the inner membrane localization for receiving upstream stimuli or further interaction with another protein involved in c-di-GMP signaling.

The interplay between c-di-GMP signaling and motility occurs at multiple levels regulation (Chen et al., 2025). CrdA significantly represses bacterial swimming motility, which is partially relieved by deletion of YcgR. Flagellar baker protein YcgR is a PilZ domain effector that responds to elevated c-di-GMP and interacts with motor proteins MotA and FliG to regulate the flagellar rotation speed and direction (Chen et al., 2025; Han et al., 2023). We also tested the functional relationship between CrdA and another c-di-GMP effector YajQ, which regulates the transcription of flagellar gene in *Salmonella* and *Xanthomonas. Campestris* (Dai et al., 2024; Jia et al., 2025). However, deletion of *yajQ* did not relieved the repression of CrdA on swimming motility. Therefore, it is possible that the YcgR protein responses to the elevated c-di-GMP produced by CrdA to regulate bacterial motility in *C. malenaticus*, though no direct interaction was observed between these two proteins (Supplementary Figure 4). Our data revealed that deletion of *ycgR* only partially relieved the repression of CrdA on bacterial motility. Meanwhile, CrdA also represses the transcription of flagellar genes. This implies that at least one additional c-di-GMP effector likely contributes to the CrdA-mediated transcriptional regulation of flagellar genes, which needs further investigation.

Cellulose is an extracellular linear glucose polymer which is frequently found in several bacterial biofilms. BcsA polymerizes UDP-D-glucose into cellulose via β-1,4-glycosidic linkages, leading to bacterial cellulose production (Richter et al., 2020). Intracellular accumulation of c-di-GMP allosterically activates BcsA through binding to its PilZ domain, thereby releasing an auto-inhibited state (Morgan et al., 2014). In *E. coli*, DgcC and PdeK directly interact with cellulose synthase subunit BcsB, establishing a local c-di-GMP signaling that control the production of cellulose (Richter et al., 2020). In this study, our data revealed that overexpression of *dgcC* also increased cellulose production in *C. malonaticus* (Figure 1e). Additionally, DGC CrdA regulates both cellulose production and biofilm formation, with its regulatory role dependent on cellulose synthase BcsA (Figure 5). No direct interaction was determined between CrdA and BcsA or BcsB. These data implies that CrdA regulates cellulose production and thus biofilm formation likely through impacting intracellular c-di-GMP pool.

Adherence to host cells and subsequent tissue colonization is often a crucial first step in establishing infection. To establish attachment on the biotic or abiotic surfaces, bacteria often produce adhesins that facilitate binding to solid substrate. A key signal of this process is the second messenger c-di-GMP, which controls adhesion production in different bacteria. Among the reported adhesins, EPS and fimbriae are the most prevalent (Lamprecht et al., 2023; Liang, 2015). Our results revealed that deletion of *crdA* decreased cellulose production and biofilm formation in *C. malonaticus* (Supplementary Figures 2b-d, 3). In accordance with this, deletion of *crdA* led to significant reduction in *C. malonaticus* adherence to intestinal epithelial cells and *in vivo* intestinal colonization (Figure 6). These results are consistent with previous studies highlighting the function of c-di-GMP signaling in modulating host cell attachment in pathogenic and probiotic bacteria (Guo et al., 2025; Hsu et al., 2025). Interestingly, our data showed that the expression of *crdA* was significant induced upon adhesion to host cells. Collectively, these data support a model that upon initial contact with host cells, induction of *crdA* expression switches bacterial cells from swimming to sessile state by repressing bacterial motility and promoting of EPS production, to facilitate successful adhesion (Figure 8).

**FIGURE 8:**
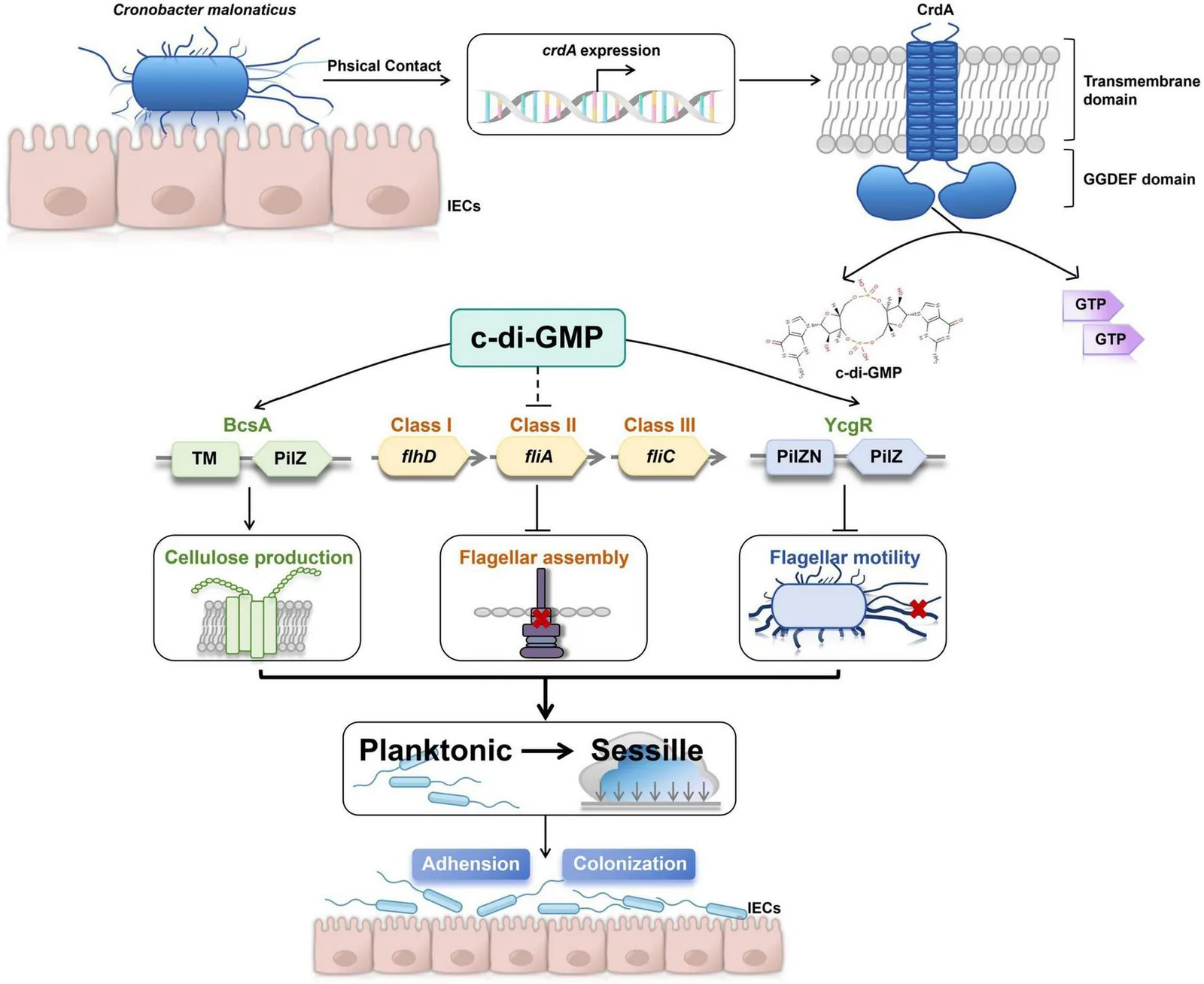
Proposed model depicting the role of CrdA in cell adhesion and intestinal colonization of *C. malonaticus*. Upon initial contact with host cells [unknown external signal(s)], induction of *crdA* expression switches bacterial cells from swimming to sessile state by repressing bacterial motility and promoting of EPS production through c-di-GMP and downstream effectors, to facilitate successful adhesion and gut colonization.

In this study, phenotypic screening in *C. malonaticus* identified six DGCs. CrdA was identified as a novel DGC that modulates c-di-GMP homeostasis, motility, biofilm formation, cell adhesion and intestinal colonization. The N-terminus of CrdA is essential for its dimerization and enzymatic activity. By governing c-di-GMP homeostasis, CrdA coordinates a phenotypic switch in bacteria: it suppresses motility (through YcgR and probably another unknown effector) and simultaneously stimulates cellulose synthesis, which in turn enhances biofilm formation, cell adhesion, and intestinal colonization. Based on the data from this study, a model depicting the role of CrdA in intestinal colonization was proposed (Figure 8). These findings expands our general understanding of c-di-GMP signaling in *C. malonaticus*, and highlight CrdA as a potential target for novel antibiofilm strategies, potentially disrupting *Cronobacter* persistence in food supply chains.

## Data Availability

The datasets presented in this study can be found in online repositories. The names of the repository/repositories and accession number(s) can be found in the article/Supplementary material.
