# Supplementary material for: The diguanylate cyclase CrdA contributes to cell adhesion and gut colonization by switching motility and sessile state of Cronobacter malonaticus
Source: Front Microbiol. 2026 Sep 25;17:1922582. doi: 10.3389/fmicb.2026.1922582 (PMC13649972; doi:10.3389/fmicb.2026.1922582)
Supplement: Supplementary file 1 [file Data_Sheet_1.docx]

Supplementary Material

# 1 Supplementary Figures and Tables

## 1.1 Supplementary Figures

**Supplementary Figure 1.** Cellulose production assay of the Δ*csgBA*, Δ*bcsA* and Δ*bcsA*Δ*csgBA* strains. Colony morphology of strains after 5 days of growth on Congo red agar plates at 28°C.

**Supplementary Figure 2.** Growth curves of the WT/EV, Δ*crdA*/EV, and Δ*crdA*/p-*crdA* strains. Overnight cultures were subcultured, and OD_600_ was determined at 2 h intervals for 14 h. WT refers to the wild-type *C. malonaticus* strain NWU01. EV denotes the empty pACYCTac plasmid transformed into the WT strain. p-*crdA* represent the overexpression construct pACYCTac-*crdA*. Error bars indicate standard deviation (SD), n = 3.

**Supplementary Figure 3.** Cellulose production assay of strains overexpressing point-mutated and truncated versions of the *crdA* gene. Colony morphology of strains after 5 days of growth on Congo red agar plates at 28°C.

**Supplementary Figure 4.** Interaction of CrdA and YcgR were identified via bacterial two-hybrid assay. The positive control (Zip-Zip) yielded blue colonies, while the negative control produced white colonies.

**Supplementary Figure 5.** YajQ is not involved in CrdA-mediated regulation of bacterial motility. Quantitative determination and statistical analysis of colony migration diameter in strains incubated on motility agar at 30°C for 6 hours. WT refers to the wild-type *C. malonaticus* strain NWU01. EV denotes the empty pACYCTac plasmid transformed into the WT strain. p-*crdA* represent the overexpression construct pACYCTac-*crdA*. Error bars indicate standard deviation (SD), n = 3. ***P* < 0.01 and **P* < 0.05 denote statistical significance.

**Supplementary Figure 6.** Interaction between CrdA and either BcsA or BcsB were identified via bacterial two-hybrid assay. The positive control (Zip-Zip) yielded blue colonies, while the negative control produced white colonies.

## 1.2 Supplementary Tables

**Supplementary Table 1.** Bacterial strains and plasmids used in this study.

| **Strains** | **Genotype or characteristics** | **Sources** |
| --- | --- | --- |
| *Escherichia coli* DH5α | F–j80*lacZ* DM15 D(*lacZYA*-*argF*)U169 *recA*1 *endA*1 *hsdR*17(rk–, mk+)*phoA* supE44 thi-1 *gyrA*96 *relA*1 *tonA* | Invitrogen |
| *Escherichia coli* S17 λ-pir | λ-pir lysogen of S–171, *thi pro hsdR* *hsdM*+ *recA* RP4 2-Tc::MuKm::Tn | AngYuBio |
| *Escherichia coli* BTH101 | A non-reverting adenylate cyclase deficient (*cya*) strain | This lab |
| *Cronobacter malonaticus* NWU01 | *C. Malonaticus* wild type | This lab |
| *Cronobacter malonaticus* Δ*crdA* | *Cronobacter malonaticus*  *crdA* deletion mutant | This study |
| *Cronobacter malonaticus* Δ*bcsA* | *Cronobacter malonaticus*  *bcsA* deletion mutant | This study |
| *Cronobacter malonaticus* Δ*csgAB* | *Cronobacter malonaticus*  *csgAB* deletion mutant | This study |
| *Cronobacter malonaticus* Δ*bcsA*Δ*csgAB* | *Cronobacter malonaticus*  *bcsA&csgAB* deletion mutant | This study |
| *Cronobacter malonaticus* Δ*ycgR* | *Cronobacter malonaticus*  *ycgR* deletion mutant | This study |
| *Cronobacter malonaticus* Δ*yajQ* | *Cronobacter malonaticus*  *yajQ* deletion mutant | This study |
| *Cronobacter malonaticus* Δ*fliC* | *Cronobacter malonaticus*  *fliC* deletion mutant | This study |
| **Plasmids** | | |
| pCVD442 | Suicide vector with a *sacB* gene, Amp^R^ | This lab |
| pACYCTac | Gene replacement vector, Amp^R^ | This lab |
| pUT18 | ColE1 origin of replication, T18 fragment, Amp^R^ | This lab |
| pKNT25 | p15A origin of replication, T25 fragment, Kan^R^ | This lab |
| p-*crdA* | pACYCTac carrying *crdA* (A_02220) | This study |
| p-*crdB* | pACYCTac carrying *crdB* (A_17775) | This study |
| p-*dgcC* | pACYCTac carrying *dgcC* (A_10090) | This study |
| p-*dgcZ* | pACYCTac carrying *dgcZ*(A_18325) | This study |
| p-*dgcQ* | pACYCTac carrying *dgcQ* (A_07560) | This study |
| p-*crdC* | pACYCTac carrying *crdC* (A_10095) | This study |
| p-*dgcF* | pACYCTac carrying *crdC* (A_10085) | This study |
| p-*crdD* | pACYCTac carrying *crdD* (A_07500) | This study |
| p-*crdE* | pACYCTac carrying *crdE* (A_17840) | This study |
| p-*crdF* | pACYCTac carrying *crdF*(A_17845) | This study |
| p-*crdG* | pACYCTac carrying *crdG* (A_01295) | This study |
| p-*crdH* | pACYCTac carrying *crdH* (A_06940) | This study |
| p-*crdI* | pACYCTac carrying *crdI* (A_20815) | This study |

**Supplementary Table 2.** Primers used in this study.

| Primers for gene overexpression | |
| --- | --- |
| *crdA-*F | ACAATTTCACACAGGAAACAATGTCAGAAAGAGATCGCGA |
| *crdA*-R | TCATCGTCGTCCTTGTAGTCATCGGCGATAACCAGACG |
| *crdB-F* | ACAATTTCACACAGGAAACAATGGATATGACTGCAAATAACTGG |
| *crdB*-R | TCATCGTCGTCCTTGTAGTCTGCGGCGCGCCGGGC |
| *dgcC*-F | ACAATTTCACACAGGAAACAATGAAGAGCGCATCTGAGC |
| *dgcC*-R | TCATCGTCGTCCTTGTAGTCAAGCGCGTAAATACGATTTT |
| *dgcZ*-F | ACAATTTCACACAGGAAACAATGGATTTGCAACGGATAG |
| *dgcZ*-R | TCATCGTCGTCCTTGTAGTCTTTCAGCGTTTCCCGC |
| *dgcQ*-F | ACAATTTCACACAGGAAACAGTGCCTGACAAGTTACTCATCA |
| *dgcQ*-R | TCATCGTCGTCCTTGTAGTCCCCTTCGTCACACACCTG |
| *crdC*-F | ACAATTTCACACAGGAAACAGGATCCATGAAAACACCCGATATCCCG |
| *dgcF*-F | ACAATTTCACACAGGAAACAATGTTTAACCAGGCGAAGC |
| *dgcF*-R | TCATCGTCGTCCTTGTAGTCGGCGCAGCAGCTCTCG |
| *crdD*-F | ACAATTTCACACAGGAAACAGGATCCATGCAACGCGGGCAGGAT |
| *crdD*-R | TCATCGTCGTCCTTGTAGTCAAGCTTTGCCAGACGTACCGCGC |
| *crdE*-F | ACAATTTCACACAGGAAACAGGATCCATGAAATTTCCTGGTATCCC |
| *crdE*-R | TCATCGTCGTCCTTGTAGTCAAGCTTGCCGATCCGCTTTCTG |
| *crdF*-F | ACAATTTCACACAGGAAACAGGATCCATGACCAACATTGCCTATGGCG |
| *crdF*-R | TCATCGTCGTCCTTGTAGTCAAGCTTGGCTCCCGGCTTCAGTC |
| *crdG*-F | ACAATTTCACACAGGAAACATTGCGCAAGGCAATATCC |
| *crdG*-R | TCATCGTCGTCCTTGTAGTCCGCGTGATAGGGGGCG |
| *crdH*-F | ACAATTTCACACAGGAAACAATGATTATTGTAGCGCTCACG |
| *crdH*-R | TCATCGTCGTCCTTGTAGTCATTTGTCGCGTCAACCTTT |
| *crdI*-F | ACAATTTCACACAGGAAACAGTGACTCTCAGGCAGTTTATTT |
| *crdI*-R | TCATCGTCGTCCTTGTAGTCAGCCGCATAAATCGATTTA |
| Primers for gene deletion | |
| pCVD442-F | GATATCGCATGCGGTACCT |
| pCVD442-R | TCCCGGGAATTCCACAA |
| *crdA*-H1-F | TTGTGGAATTCCCGGGACCATGGTTTCCTGAAGCCGTCTCG |
| *crdA*-H1-R | ATCGGCGATAACCAGACGGGGCTAACCTGCACTGTG |
| *crdA*-H2-F | CACAGTGCAGGTTAGCCCCGTCTGGTTATCGCCGAT |
| *crdA*-H2-R | GGTACCGCATGCGATATCCTCGAGGGACACGTTAAACGTGCG |
| *bcsA*-H1-F | TTGTGGAATTCCCGGGATTAGCATGCAAACCGGGAT |
| *bcsA*-H1-R | TTTTCTTTTCATCGCATATTCAGCGCCCTCACTCCCGTA |
| *bcsA*-H2-F | TACGGGAGTGAGGGCGCTGAATATGCGATGAAAAGAAA |
| *bcsA*-H2-R | AGGTACCGCATGCGATATCTCACCAGCAATTTGACCCA |
| *csgAB*-H1-F | TTTGTGGAATTCCCGGGATCTGTCGCGTAAAATACGCC |
| *csgAB*-H1-R | GGCCCTGCCCAGGATAAATTACTCCTGGCCCTTAAGGC |
| *csgAB*-H2-F | GCCTTAAGGGCCAGGAGTAATTTATCCTGGGCAGGGCC |
| *csgAB*-H2-R | AGGTACCGCATGCGATATCATGAATAGCGCCATCCACAC |
| *ycgR*-H1-F | TTGTGGAATTCCCGGGACCATGGGAACCGGCAGGCAAAGTG |
| *ycgR*-H1-R | GCGATGAACGCCATGTAAGTCGCGTGATTCCTCAAATG |
| *ycgR*-H2-F | CATTTGAGGAATCACGCGACTTACATGGCGTTCATCGC |
| *ycgR*-H2-R | AGGTACCGCATGCGATATCCTCGAGTTCGGATCAAAACGCTCC |
| *fliC*-H1-F | ACAGCTATGACCATGATTACGAATTCAGCCGATAATCCCCTGCA |
| *fliC*-H1-R | TTTTGAAAGCTTGAGCGTCTACTCGAGAATTTAATTCCTTCAAAAATTGG |
| *fliC*-H2-F | CCAATTTTTGAAGGAATTAAATTCTCGAGTAGACGCTCAAGCTTTCAAAA |
| *fliC*-H2-R | GCCTGCAGGTCGACTCTAGAGGATCCATCGCAGGAGACGAGTACC |
| *yajQ*-H1-F | ATTGAATTCAGTGCCGATGCGCTCATACT |
| *yajQ*-H1-R | CGGTTCGGCTGCCGCGTTTTCTATCTCCCTCACAGTATGT |
| *yajQ*-H2-F | ACATACTGTGAGGGAGATAGAAAACGCGGCAGCCGAACCG |
| *yajQ*-H2-R | ATTGGATCCGTTTATCGGCGTCAGCTTTGG |
| *yajQ*-F | ACAATTTCACACAGGAAACAATGCCATCTTTTGACATTGTCTC |
| *yajQ*-R | TCATCGTCGTCCTTGTAGTCGTCGCGGAAGTTGTTGAACT |
| Primers for bacterial two-hybrid system | |
| *crdA*-T18-F | CCTGCAGGTCGACTCTAGAGATGTCAGAAAGAGATCGCGA |
| *crdA*-T18-R | TTCGAGCTCGGTACCCGGGGATCGGCGATAACCAGACG |
| *crdA*-T25-F | CCTGCAGGTCGACTCTAGAGATGTCAGAAAGAGATCGCGA |
| *crdA*-T25-R | TTCGAGCTCGGTACCCGGGGATCGGCGATAACCAGACG |
| *bcsA*-T25-F | CATTCTAGAGATGAAAAGAAAACTCTCCTGGAT |
| *bcsA*-T25-R | CATCCCGGGTCATTGTTGAGCCATAACCG |
| *bcsB*-T25-F | CATTCTAGAG ATGAAAAGAAAACTCTCCTGGAT |
| *bcsB*-T25-R | CATCCCGGGTTACTCATGGTTGCCCGG |
| *ycgR*-T25-F | CATTCTAGAGATGAGCAATTACAGTGAGCAGTTTC |
| *ycgR*-T25-R | CATGGATCCTCGCGCACCCGGCTGGC |
| *crdA*^173-348^-T18-F | CACTGCAGGTCGACTCTAGACGCGACAGCCTGACGA |
| *crdA*^173-348^-T18-R | AATTCGAGCTCGGTACCCGG TTAATCGGCGATAACCAGAC |
| *crdA*^173-348^-T25-F | GCTGCAGGGTCGACTCTAGACGCGACAGCCTGACGA |
| *crdA*^173-348^-T25-R | TTACTTACTTAGGTACCCGG TTAATCGGCGATAACCAGAC |
| *crdA*^M1^-T18-F | CAT AAGCTTG ATGTCAGAAAGAGATCGCGA |
| *crdA*^M1^-T18-R | CAT GGATCCTC ATCGGCGATAACCAGACG |
| *crdA*^M1^-T25-F | CAT AAGCTTG ATGTCAGAAAGAGATCGCGA |
| *crdA*^M1^-T25-R | CAT GGATCCTC ATCGGCGATAACCAGACG |
| Primers for gene mutation and truncation | |
| *crdA-*M-F | GCGACATCGTGGCGCGCTGGGCCGCCGAAGAGTTTCTGGTGCTGAT |
| *crdA-*M-R | ATCAGCACCAGAAACTCTTCGGCGGCCCAGCGCGCCACGATGTCGC |
| *crdA*^173-348^-F | ACAATTTCACACAGGAAACACGTTCGGTAGAATTCACCC |
| *crdA-*M-1-F | CTTGGCACTGTGGGGGCCGCCCTGGCCCATCACACCGGTACG |
| *crdA-*M-1-R | CGTACCGGTGTGATGGGCCAGGGCGGCCCCCACAGTGCCAAG |
| *crdA-*M-2-F | CTACGGTGTTGCTGGGCTGGGCGAGTAATTTTTATCTGTTTGT |
| *crdA-*M-2-R | ACAAACAGATAAAAATTACTCGCCCAGCCCAGCAACACCGTAG |
| *crdA-*M-3-F | GGTTAAGCCCGGCAGTGCTGGCAGGCATCGGTGTGGCTAATGC |
| *crdA-*M-3-R | GCATTAGCCACACCGATGCCTGCCAGCACTGCCGGGCTTAACC |
| *crdA-*M^21-348^-F | ACAATTTCACACAGGAAACAATTATTCCGATCGTCTGGCT |
| Primers for qRT-PCR | |
| *gyrB*-F | TTGGCTGCGGTATTGGT |
| *gyrB*-R | GGCATCTGACGGTAGAAGAA |
| *flhD*-F | TATCTACGACATCAACCT |
| *flhD*-R | CATCTCTTCGCTAATACC |
| *fliA*-F | AAGAATATCGTCAGATGTT |
| *fliA*-R | ATTCAATCGCTTCCATAA |
| *fliC*-F | GAACAACTCTATCAACAAC |
| *fliC*-R | TGAGACAGTACAGAAGTA |
| *ompR*-F | ATTCCTAAACCGTTCAAC |
| *ompR*-R | GATAATGGCTTCTTCCTG |
| *ompA*-F | TATAAAGGCGACACTGTA |
| *ompA*-R | ATGTTAGAAGAGGAGTCA |
| *ompC*-F | TACTTCTCTGATAACAACA |
| *ompC*-R | AATCTGATATTCCCACTG |
| *ompX*-F | TGAAATACCGTTATGAGTT |
| *ompX*-R | TTACCATATACGCCATCT |
| *uvrY*-F | ACGCATACTTGAAGATAT |
| *uvrY-*R | TTAATGTCTGAAGAATAACG |
| *luxR-*F | CGCAGAACATGGAATATG |
| *luxR-*R | GTCCTGTCAGATAATTGG |
| *wzx*-F | GGTGGATAATTCAGATAGC |
| *wzx*-R | CCTGTTAATCCGTAGTTC |
| *flgJ*-F | TCAGATGGAAGGGATGTT |
| *flgJ*-R | TGATCGTACATGCTGGTA |
| *sodA-*F | CGTACTATCTGAAATTCCA |
| *sodA-*R | TTATTTCTGAGAAGCGAA |
| *lpxB-*F | AATGTATGCTGGCGAAAT |
| *lpxB-*R | AGCGACACAAATTCAGTT |
| *rpoS*-F | TTGATGAGAACGGAGTAG |
| *rpoS*-R | TTAGCAGAGGCGAATAAC |
| *rpoN*-F | ATATGTTATTCCCGATGTC |
| *rpoN*-R | CGTACTGCTGATTGATTT |
| *fur*-F | GACAACAATACCGCATTA |
| *fur*-R | TCAATCAGACGCTTATAGA |
| *groES*-F | GATCGTCAAGCGTAAAGA |
| *groES*-R | CCATAGCCATCGTTGAAA |
| *osmY*-F | GAAAGCACCAAATCTACC |
| *osmY*-R | ATCTGTTCGTCATCTACC |
| *mflA*-F | GGAATCGGTAAAGAAAGC |
| *mflA*-R | GTTGTCGGTTTGGATTAC |
| *cpxR*-F | CTTGACCTCCTGGATGAC |
| *cpxR*-F | CATAATAACGGGTGTCTGG |

**Supplementary Table 3.** Hydrogen bond interactions between homodimers of CrdA.

| **NO.** | **Chain A** | **Chain B** |
| --- | --- | --- |
| 1 | Y135 | E95 |
| 2 | Y135 | N153 |
| 3 | R138 | N153 |
| 4 | T178 | T178 |
| 5 | K182 | L9 |
| 6 | K188 | R201 |
| 7 | R191 | R201 |
| 8 | R191 | D5 |
| 9 | Y198 | D5 |
| 10 | Q206 | Q8 |
